# Supraspinatus Muscle Degeneration, Inflammation, and Regeneration Vary By Location in a Rat Model of Severe Rotator Cuff Tear

**DOI:** 10.1007/s40883-024-00343-3

**Published:** 2026-03-19

**Authors:** Molly E. Ogle, Kasheena Box, Keshav R. Shah, Johnna S. Temenoff

**Affiliations:** 1 Coulter Department of Biomedical Engineering, Georgia Institute of Technology and Emory University, Atlanta, GA 30332 USA; 2 Petit Institute of Bioengineering and Bioscience, Georgia Institute of Technology, Atlanta, GA 30332 USA

**Keywords:** Rotator cuff, Supraspinatus muscle, Supraspinatus tendon, Inflammation, Degeneration

## Abstract

**Purpose:**

Degeneration of the human supraspinatus muscle after rotator cuff tendon tear varies by location within the muscle; however, the localization of changes to muscle fiber size, amount of regenerating fibers, and cellular immune response have not been previously characterized. Due to the proximity to the injured tendon, we hypothesized that these processes are more pronounced near the lateral myotendinous junction region of the muscle and the intramuscular tendon.

**Methods:**

Spatially-defined immunohistochemical analysis was used to evaluate lengthwise and radial differences within the supraspinatus muscle in a rat model of full supraspinatus and infraspinatus tendon transection and subscapular denervation. Muscle fiber diameter was quantified, regeneration was evaluated by myofiber central nuclei and embryonic myosin heavy chain, and the immune environment was assessed by the presence of macrophage subsets and T cells.

**Results:**

Degeneration (smaller fiber diameter), regeneration (embryonic myosin heavy chain-positive fibers), and macrophage infiltration are more substantial in the lateral muscle near the myotendinous junction and radially near the intramuscular tendon. The muscle adjacent to the myotendinous junction had a balanced M1-like and M2-like macrophage response up to 2 weeks post-injury followed by an M1-dominant inflammation. The medial muscle belly maintained an M1-dominant response throughout 3 weeks. T cells were not highly abundant, however, were most elevated one week after injury near the intramuscular tendon. Fibrous infiltration was observed 1-week post-injury throughout the muscle, while fatty infiltration was elevated after 3 weeks only in the lateral muscle region.

**Conclusions:**

Together, these findings suggest that the supraspinatus muscle undergoes regional changes (particularly muscle fiber size, markers of regeneration, and macrophage infiltration) after supraspinatus tendon injury.

**Lay Summary and Future Work:**

Rotator cuff tendon tear in the rat shoulder leads to a greater inflammatory response and a greater early pro-regenerative response in the supraspinatus muscle spatially near the muscle–tendon attachment compared with areas deeper in the muscle in the first two weeks after the tendon is injured. Both fatty and fibrous muscle degeneration were observed over three weeks throughout the muscle, with less spatial localization. Together, these data indicate that there are regional differences in damage and repair of the supraspinatus muscle early post-injury. These findings may help interpret results from clinical studies of muscle degeneration after rotator cuff tear and may lead to improved, spatially-targeted approaches for delivery of therapeutics to muscle after rotator cuff tendon tear.

**Supplementary Information:**

The online version contains supplementary material available at 10.1007/s40883-024-00343-3.

## Introduction

Rotator cuff injuries affect a large proportion of the adult population, and the incidence increases with age [1]. Rotator cuff tear (RCT) is characterized by the tear of one or more of the four tendons making up the rotator cuff, the supraspinatus tendon being the most frequently damaged [2]. The supraspinatus and infraspinatus muscles of the rotator cuff are subject to mechanical unloading and nerve impingement after RCT followed by an inflammatory, fibrous, and fatty response within the muscle [3, 4]. Muscle pathology secondary to RCT is highly correlated with failure of surgical repairs, suggesting that extent of muscle degeneration contributes to the success of surgical repair and therefore the ability of the joint to heal [5, 6]. While features of the pathological process are well known, the signals and sequence of events driving these changes are not fully characterized, particularly the regional progression of muscle degeneration within the supraspinatus muscle following injury.

Clinical observations have shown that human supraspinatus muscle atrophy and fatty infiltration can progress asymmetrically within the muscle [7–9]. Further evidence shows that there are spatially patterned metabolic and gene expression changes in muscle tissue after RCT in human patients [9, 10]. In murine severe RCT, fibrosis begins in the muscle region closest to the tendon enthesis at the humeral head (lateral) and progresses over time through the length of the muscle [11] suggesting that muscle degeneration may be spatially defined. However, many pre-clinical studies utilize histological analysis that captures only a small random sample of a muscle cross-section and does not reveal spatial differences within the muscle. Therefore, a critical gap remains in knowledge regarding the spatial organization of muscle degenerative events following RCT, which may hold critical insights into the mechanisms of disease progression.

Therefore, the goal of this study was to characterize myofiber damage, regeneration, fibrosis, fatty infiltration, and inflammation, in discrete regions of the supraspinatus muscle. Specifically, we hypothesized that measures of muscle damage, inflammation, and repair would be more pronounced near the lateral MTJ region of the muscle and near the intramuscular tendon that stretches from the greater tuberosity of the humeral head through the muscle belly [12, 13] (representing regions closer to the initially-injured tendon and the subacromial bursa) [14]. To spatially evaluate injury metrics, both lengthwise and radial/cross-sectional regions of interest were defined for comparisons in immunohistochemical analysis of the supraspinatus muscle. Along the length of the muscle, two regions were evaluated, the most lateral region (MTJ), and the middle of the muscle (MB). To determine whether muscle tissue associated with the intramuscular tendon was differentially affected after RCT, this study also compared muscle radially near or far from the intramuscular tendon. Degeneration was assessed by measuring changes in myofiber cross-sectional area (laminin staining), while regeneration was assessed by myofiber expression of embryonic myosin heavy chain (eMHC) and the presence of centrally-located nuclei. Fibrosis was measured by immunohistochemical staining of collagen and fatty infiltration by evaluation of cells expressing perilipin. Adaptive and innate immune cell infiltration were analyzed by staining for M1-like inflammatory macrophages (CD86) and M2a-like pro-regenerative macrophages (CD206) and T-cells (CD3).

## Materials and Methods

### Rotator Cuff Injury Model

All animal procedures received prior approval by the Georgia Institute of Technology Institutional Animal Care and Use Committee. Surgical rotator cuff injury was implemented as previously described [15]. Sprague–Dawley rats (Charles River, CD1) 8 weeks of age were anesthetized with isoflurane. A skin incision over the left deltoid, followed by an incision in-line with the deltoid fibers to expose the humeral head. A 2 mm suprascapular nerve segment was resected from beneath the deltoid. The supraspinatus and infraspinatus tendons were isolated at the humeral head and tagged with a 4–0 silk suture (Oasis, MV-683-V) prior to transection. A 2 mm piece of sterile PharMed BPT biocompatible tubing (Saint-Gobain) was slipped over the transected end of the tendon and sutured in place to reduce tendon re-attachment. Deltoid muscle and skin were each rejoined with Vicryl 4–0 absorbable sutures (Ethicon) and wound clips, respectively. A slurry of powdered Metronidazole (Unichem) and Liquid Bandage (New Skin) was applied over the wound and sustained release Buprenorphine was injected subcutaneously for analgesia. Animals were allowed normal cage movement following surgery. Animals were humanely euthanized after 7 (n = 6), 14 (n = 5), or 21 (n = 5) days.

### Sample Histology and Immunohistochemistry

**Fig. 1:**
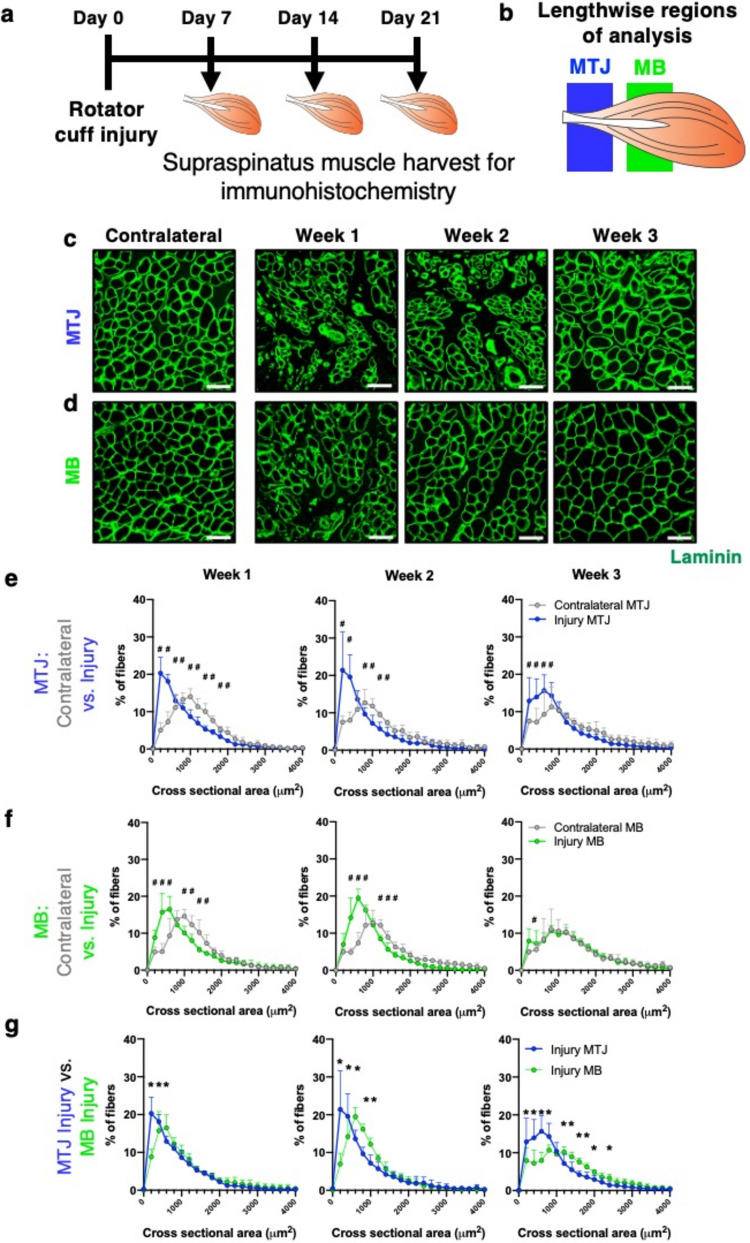
Increase in smaller myofibers is most prominent at the myotendinous junction (**a**) Supraspinatus muscle was harvested 1, 2, or 3 weeks after rotator cuff injury. (**b**) Muscle sections from the myotendinous junction (MTJ) area (Muscle approx. 0-1000um from union of tendon and muscle) and an area deeper in the muscle belly (MB) (~ 2000-3000um from union of tendon and muscle) were analyzed. (**c-d**) Representative images of muscle fiber borders (laminin, green) near the intramuscular tendon in the (**c**) myotendinous junction (MTJ) and the (**d**) muscle belly (MB). (**e–f**) Distribution of myofiber cross-sectional area in the (**e**) MTJ or (**f**) MB. (**g**) Comparison of cross-sectional area between MTJ and MB regions. (2-way ANOVA with post-hoc multiple comparisons tests, # p < 0.05 vs. contralateral; *p < 0.05 MTJ vs. MB; n = 5–6; scale bar 50 μm)

Supraspinatus muscle was harvested bilaterally on days 7, 14, and 21, with contralateral as internal control (n = 5–7 animals/timepoint) (Fig. 1a). Upon harvest of injured and contralateral muscles, muscles were weighed to assess gross atrophy. Muscles were submerged in phosphate buffered saline (PBS, Corning) with 10% OCT (Tissue-Plus) (v/v) and either 0%, 10%, or 20% sucrose (w/v) for 20 min each under vacuum. Muscles were covered with OCT and incubated overnight under vacuum prior to freezing in a pre-chilled bath of hexanes (Sigma) surrounded by dry ice-cooled ethanol (Sigma). Muscles were sectioned (10 μm) on a CryoStar NX70 cryostat from the tendon–muscle interface. Thereafter, sections were collected at regular intervals 20–50 μm within the first 3.5 mm segment of the distal aspect of the muscle. For all analyses, two sections were randomly selected from the first 0–1.5 mm lateral segment (MTJ) and two sections were selected from 2.5–3.5 mm segment (MB) (Fig. 1b).

Immunohistochemical staining was performed for laminin in the myofiber basal lamina to measure cross-sectional area (Sigma); eMHC to measure early myofiber regeneration (DSHB); collagen I to visualize fibrous infiltration (Abcam); perilipin to evaluate fatty infiltration (Cell Signaling Technology, 9349S); macrophage surface markers CD86 (Bio-Rad, MCA2874T) and CD206 (Abcam, ab16669) for M1-like and M2-like phenotype macrophages, respectively; and CD3 to measure all T cells (Abcam, ab16669). Slides were incubated in blocking permeabilization buffer containing 5% (w/v) bovine serum albumin, 0.5% goat serum, and 0.1% Triton-X-100 in PBS for 30 min. Primary antibody was applied in 50% blocking buffer for 1 h to overnight. Sections were washed and incubated with secondary antibodies for 30 min. Sections were incubated with Hoechst (Invitrogen) in PBS to visualize cell nuclei, washed in PBS, fixed in 4% paraformaldehyde (ThermoFisher) and mounted with Anti-fade Mounting Medium (Vectashield).

Sections were imaged on a Zeiss LSM710 confocal microscope using a 10 or 20X objective. Muscle sections were imaged using the tilescan function in Zen Black software (Zeiss) to create a mosaic image of the entire section. Images were exported as Maximum Intensity Projection of for further analysis.

### Image Analysis

For each animal, two sections were evaluated in the MTJ and two in the MB. Muscle cross-section images were evaluated in FIJI ImageJ software (NIH). To define the region radially “near” the intramuscular tendon, the outline of the tendon was identified and a 500 μm radius of surrounding tissue was defined. Beyond 500 μm from the intramuscular tendon was defined as radially “far” from the intramuscular tendon. For cross-sectional muscle fiber area analysis, laminin-stained images were inverted prior to analysis with the “Analyze Particles” tool. At least 500 total randomly selected fibers were assessed in both the near and far regions of interest; a random number generator was used to select 500 fibers for analysis. For eMHC, central nuclei, immune cell, and perilipin + cell analyses, the number of positive events were normalized by area of analysis. For fibrosis analysis, the collagen stain channel was converted to grayscale and a threshold was applied to all images. Area of collagen staining was recorded relative to the total area of analysis.

### Statistical Analysis

Sample sizes and replicates are as stated in the figure legends, error bars represent mean ± standard deviation. Myofiber cross-sectional area is displayed as a histogram of 500 randomly selected fibers per sample. Differences between groups were assessed by 2-way ANOVA with repeated measures where appropriate followed by Tukey’s or Bonferroni’s post hoc test. All analysis was performed using Prism9 (GraphPad).

## Results

### Decrease in Muscle Cross-sectional Area After Acute Rotator Cuff Injury is Most Severe in the MTJ Region

Gross atrophy (as determined by decrease in muscle mass) of the supraspinatus muscle was detected after 2 and 3 weeks; however, the muscle weight was not different from contralateral control 1 week after injury (Fig. S1). Muscle cross-sections stained with laminin showed disorganization and apparent shrinkage of the myofibers near (< 500 μm) the intramuscular tendon, particularly in the MTJ, 1 and 2 weeks after injury (Fig. 1c-d). In the injured MTJ near the intramuscular tendon, there was a significant increase in small cross-sectional area fibers (< 400 μm^2^) in weeks 1 and 2, and < 600 μm^2^ at 3 weeks post-injury relative to contralateral control (Fig. 1e, S2). In week 1, there was also a decrease in fibers between 1200–1800 μm^2^ when compared with contralateral controls (Fig. 1e, S2a). In the injured MB region near the intramuscular tendon, 1 week after injury there was an increase in myofibers with cross-sectional area < 600 μm^2^ and an increase in fibers between 200–800 μm^2^ relative to contralateral control, however, by week 3, there was no significant difference (Fig. 1f, S2b). The MTJ region had significantly more small cross-sectional area fibers (week 1 & 2: < 400 μm^2^; week 3: < 800 μm^2^) versus the MB through 3 weeks post-injury near the intramuscular tendon (Fig. 1g, S2b).

### Changes in Myofiber Cross-sectional Area Are Concentrated Near the Intramuscular Tendon

**Fig. 2:**
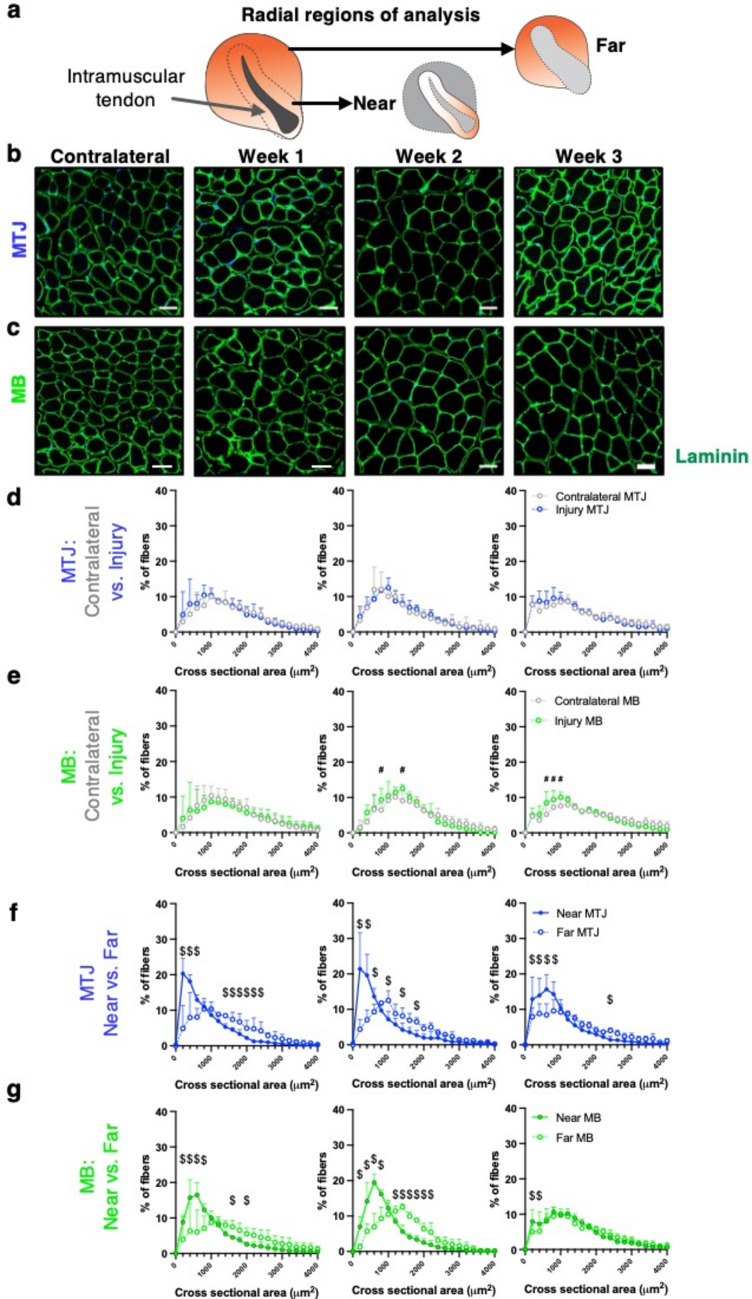
Myofiber cross-sectional area far (> 500um) from the intramuscular tendon is similar to uninjured (**a**) Each section was subdivided into two sub-areas based on their location relative to the intramuscular tendon. “Near” is < 500 μm and “Far” is > 500 μm from the intramuscular tendon. (**b-c**) Representative images of muscle fiber borders (laminin, green) > 500 μm from the intramuscular tendon in the (**b**) MTJ and the (**c**) MB. (**d-e**) Distribution of myofiber cross-sectional area far from the intramuscular tendon in the (**b**) MTJ or (**e**) MB. (**f-g**) Comparison of cross-sectional area Near or Far from the intramuscular tendon in the (**f**) MTJ and (**g**) MB regions. (2-way ANOVA with post-hoc multiple comparisons tests, # p < 0.05 vs. contralateral; $ p < 0.05 Near vs. Far; n = 5–6; scale bar 50 μm)

Regardless of the lengthwise location assessed (MTJ or MB), injured myofibers at least 500 μm (“far”) from the intramuscular tendon appeared (Fig. 2a) qualitatively more similar to contralateral fiber organization (Fig. 2b-c, S2). Quantification of fiber cross-sectional area showed fibers in the injured MTJ far from the intramuscular tendon had the same size distribution as corresponding contralateral control fibers (Fig. 2b, d). In the MB, small differences in myofiber cross-sectional area versus contralateral were noted at 2 and 3 weeks (Fig. 2c, e). Comparison of fibers near and far from the intramuscular tendon in the injured MTJ showed that there are significantly more small fibers near the intramuscular tendon compared with far (week 1 & 2: < 600 μm^2^; week 3: < 800 μm^2^) (Fig. 2f). In the MB, smaller fibers were also more abundant near the tendon than far from the tendon (week 1 & 2: < 800 μm^2^; week 3: < 400 μm^2^) (Fig. 2g). Contralateral control myofibers near the intramuscular tendon (Fig. S2a, b) did not appear qualitatively different from far from the intramuscular tendon (Fig. S2c, d) and were not different in cross-sectional area by location or over time.

### Myofiber Regeneration is Concentrated in the MTJ and Near the Intramuscular Tendon

**Fig. 3:**
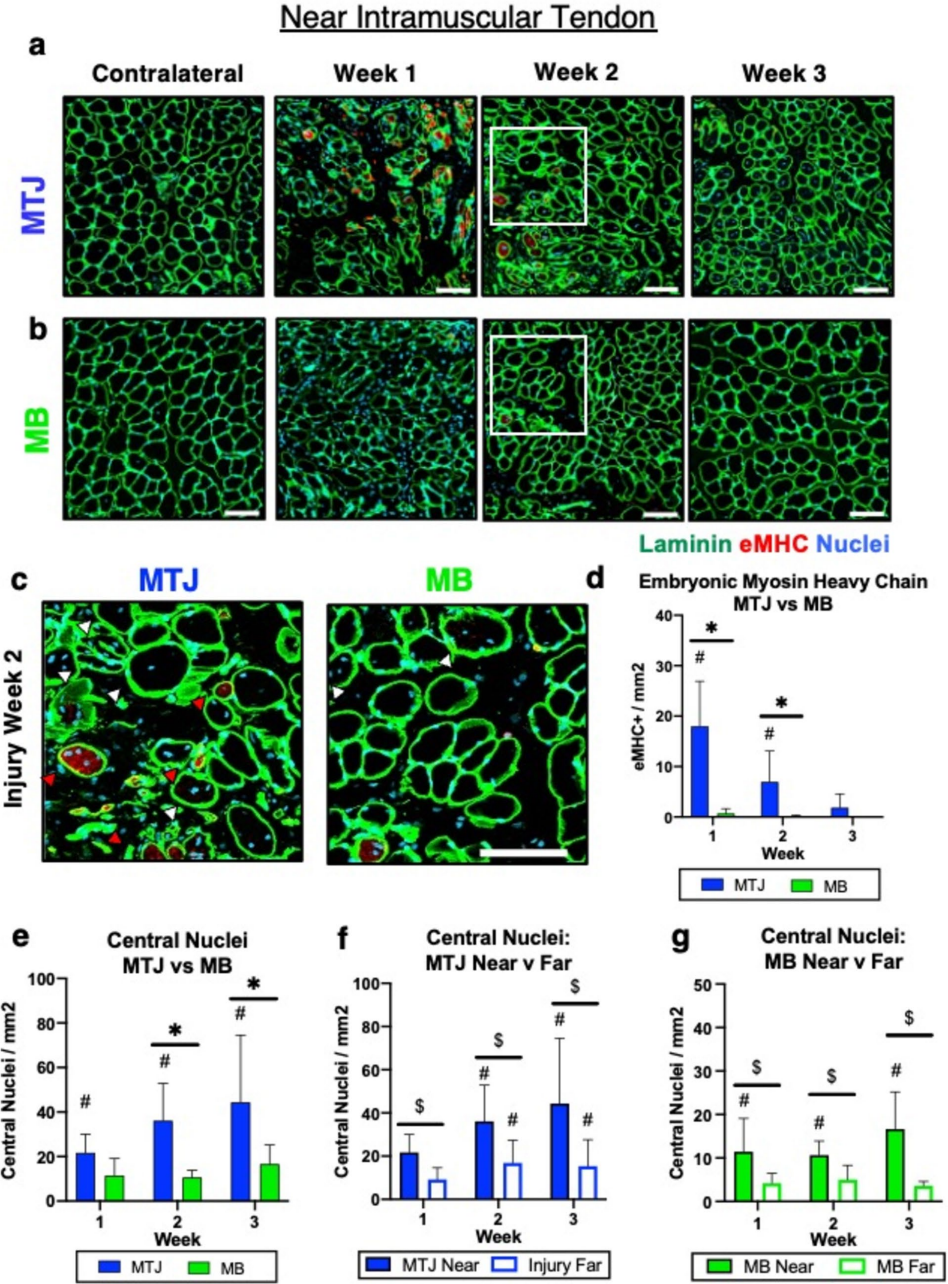
Myofiber regeneration is concentrated in the MTJ and near the intramuscular tendon (**a-b**) Representative images of regenerating myofibers near the intramuscular tendon in the (**a**) MTJ and the (**b**) MB (laminin, green; eMHC, red; nuclei, blue; scale bar, 50 μm). (**c**) Enlarged view of white boxes in **a** and **b** showing eMHC + fibers (red arrowheads) and central nuclei (white arrowheads) (scale bar 100 μm). (**d**) Quantification of early regenerating myofibers expressing eMHC in the MTJ and MB. (**e**) Quantification of regenerating muscle fibers with centrally-located myonuclei. (**f-g**) Comparison of the density of regenerating myofibers Near or Far from the intramuscular tendon in the (**f**) MTJ or (**g**) MB. (2-way ANOVA with post-hoc multiple comparisons tests, # p < 0.05 vs. contralateral; *p < 0.05 MTJ vs. MB; $ p < 0.05 Near vs. Far; n = 5–6)

A substantial population eMHC + myofibers were found near the injured intramuscular tendon in the MTJ in weeks 1 and 2 relative to contralateral control, but not in the MB region (Fig. 3a-d). Contralateral muscles did not have eMHC + myofibers at any timepoint (Fig. S3). Centrally-located myonuclei, which are indicative of regenerating fibers, were found in injured muscle, but not the contralateral muscles (Fig. 3a-c, S3). At all timepoints, relative to contralateral control, central nuclei were elevated in the MTJ near the intramuscular tendon with an increasing trend over time (Fig. 3e, S3). In weeks 2 and 3 in the MTJ region, central nuclei were elevated relative to control far from the intramuscular tendon. At all timepoints, compared with near the intramuscular tendon, there was a lower density of central nuclei far from the intramuscular tendon (Fig. 3f). In the MB, central nuclei were more prevalent near the intramuscular tendon versus far (Fig. 3g).

### Fibrous Infiltration is Elevated Throughout the Muscle As Early As One Week

**Fig. 4:**
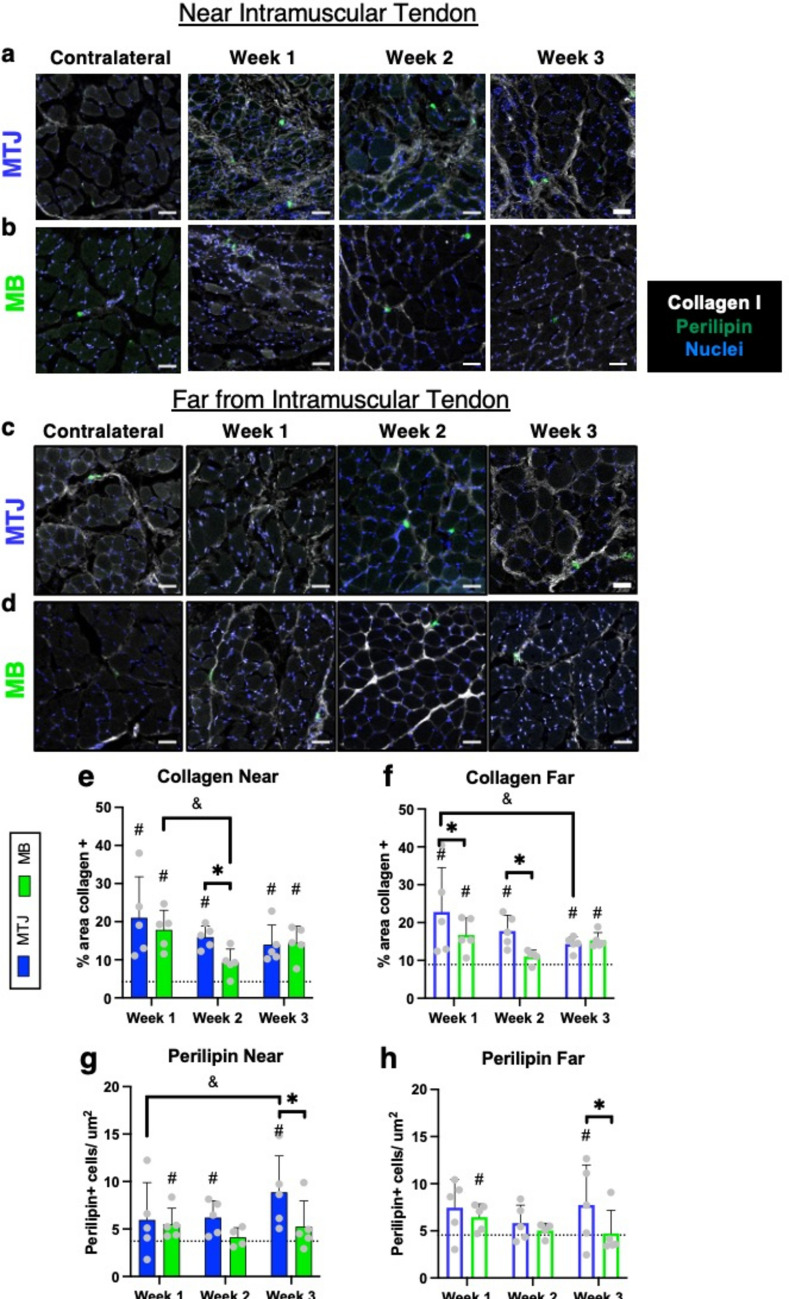
Fibrous infiltration increases over time in the MTJ and near the intramuscular tendon (**a-d**) Representative images of collagen infiltration (white) and perilipin (green) in the MTJ (**a**) near or (**c**) far from the intramuscular tendon and MB (**b**) near or (**d**) far from the intramuscular tendon. (**e–f**) Quantification of collagen (% area) (**e**) near or (**f**) far from the intramuscular tendon**.** (**g-h**) Quantification of perilipin + cells per area (**g**) near or (**h**) far from the intramuscular tendon. (2-way ANOVA with post-hoc multiple comparisons tests, # p < 0.05 vs. contralateral; *p < 0.05 MTJ vs. MB; & p < 0.05 between indicated timepoints; n = 5–6; dotted line: average of contralateral controls; scale bar 50 μm)

Collagen immunostaining was evident between myofibers throughout the injured muscle from 1 to 3 weeks, but less was observed in contralateral control muscles (Fig. 4a-d, S4). Near the intramuscular tendon, the percent area of collagen staining was significantly elevated in both the MTJ and MB compared with contralateral control (Fig. 4e). Far from the intramuscular tendon, there was a higher percent area of collagen staining in the MTJ versus the MB for the first two weeks following injury (Fig. 4f).

### Fatty Infiltration is Elevated Throughout the Muscle As Early As One Week

Immunostaining for perilipin identified several perilipin + cells between muscle fibers (Fig. 4a-d). Some perilipin + cells were present in contralateral control muscles throughout the time-course (Fig. S4). Near the intramuscular tendon, in the MTJ, perilipin + cells were significantly increased 2 and 3 weeks after injury, while in the MB, perilipin + cells were increased at week 1 (Fig. 4g, S4). Far from the intramuscular tendon, perilipin + cells were increased in week 1 in the MB and week 3 in the MTJ (Fig. 4h). By week 3, there was a higher concentration of perilipin + cells in the MTJ versus the MB (Fig. 4g-h).

### Macrophage Infiltration Varies By Inflammatory Phenotype, Location, and Time After Injury

**Fig. 5:**
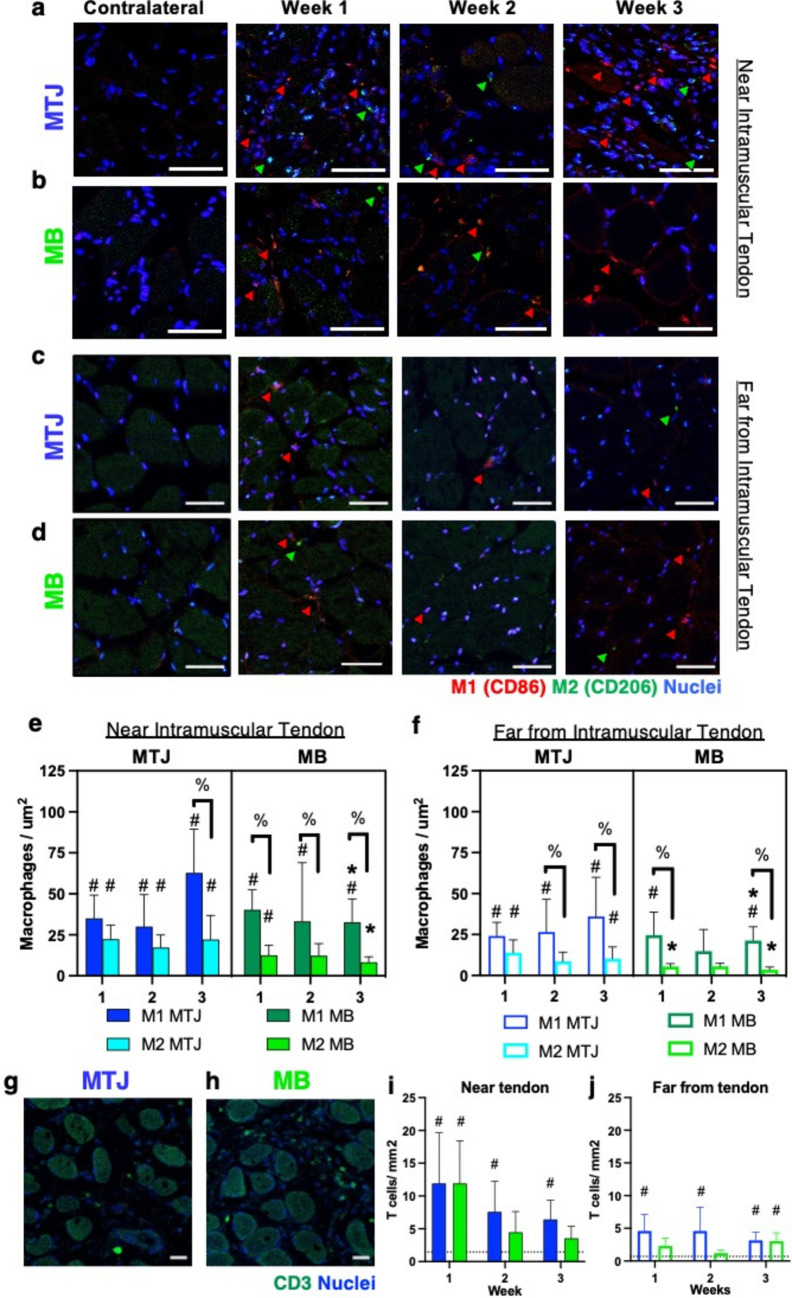
Macrophage and T cell infiltration varies by inflammatory phenotype, location, and time (**a-b**) Representative images of macrophages in the (**a**) MTJ or (**b**) MB near the intramuscular tendon (M1-like/CD86, red; M2-like/CD206, green; nuclei, blue; scale bar, 50 μm). (**c-d**) Representative images of macrophages in the (**c**) MTJ or (**d**) MB far from the intramuscular tendon (**e–f**) Comparison of M1- and M2-like phenotype macrophages (**e**) near the intramuscular tendon or (**f**) far from the intramuscular tendon. (2-way ANOVA, # p < 0.05 vs. contralateral; * p < 0.05 MTJ vs. MB; % p < 0.05 M1-like vs. M2-like; n = 5–6). (**g-h**) Representative images of T cells in the supraspinatus muscle near the intramuscular tendon in the (**g**) MTJ or (**h**) MB; (CD3, green; nuclei, blue; scale bar 20 μm). (**i-j**) Quantification of T cell infiltration (**i**) near or (**j**) far from the intramuscular tendon. (2-way ANOVA with post-hoc multiple comparisons tests, # p < 0.05 vs. contralateral; dotted line: average of contralateral controls; n = 5–6)

Inflammatory M1-like macrophages (CD86 +) were elevated at all timepoints after injury near the intramuscular tendon in both the MTJ and the MB regions compared with corresponding contralateral muscles and did not change over time (Fig. 5a-b, e, S5). Far from the intramuscular tendon, M1-like macrophages were also elevated in the MTJ and MB (Fig. 5c-d, f). By 3 weeks after injury, there were fewer M1-like macrophages in the MB region compared with MTJ regardless of location relative to intramuscular tendon (Fig. 5e-f).

Pro-regenerative M2-like macrophages (CD206 +) were increased in injured muscle near the intramuscular tendon in the MTJ at all timepoints, but in the MB, M2-like cells were only elevated at week 1 (Fig. 5a, e). In the MTJ, M2-like macrophage density was not statistically different from M1-like density for weeks 1–2, but at week 3, there were significantly more M1-like macrophages compared with M2-like (Fig. 5e). In the MB, M1-like macrophages were the dominant phenotype for all timepoints (Fig. 5e). Far from the intramuscular tendon, M2-like cells were elevated at weeks 1 and 3 in the MTJ but not in the MB (Fig. 5f). There were significantly fewer M2-like macrophages in the MB relative to MTJ throughout the muscle (Fig. 5e-f).

### T cells in the Supraspinatus Muscle Peak 1 week After Injury

T cells (CD3 +) were found between myofibers predominantly near the intramuscular tendon in injured muscle, but not contralateral controls (Fig. 5g-h, S6-7). Near the intramuscular tendon, T cells were elevated at all timepoints in the MTJ, but only at week 1 in the MB (Fig. 5i). Far from the intramuscular tendon, there was a lower number of T cells compared with near the tendon; however, there were still more CD3 + cells in the injured MTJ compared with contralateral at all timepoints (Fig. 5j).

## Discussion

The supraspinatus muscle undergoes asymmetric changes in degeneration and fatty infiltration following severe RCT in humans [7, 8, 10, 16, 17]; however, the early spatial localization of degenerative, inflammatory, and regenerative changes associated with supraspinatus muscle pathology have not previously been characterized. Through histological analysis in distinct muscle regions, the current study illustrated that fiber cross-sectional area, regeneration, and inflammation were all differentially represented in the lateral MTJ region and near the intramuscular tendon when compared with the MB or far from the intramuscular tendon, respectively. Fatty infiltration was low for the timepoints characterized in this model, but did show an increased incidence in the MTJ versus the MB after 3 weeks. Collagen fibrous infiltration was more pervasive throughout the muscle, rather than being concentrated in specific areas along the muscle length. Together, these data indicate that there are regional differences in damage and repair of the supraspinatus muscle in the early post-injury phase which may suggest differing damage/repair mechanisms governing the pathological process.

Whereas gross atrophy is a known hallmark of RCT-related muscle disease, the measurement of gross atrophy may miss more subtle degenerative changes in the muscle and does not allow for the understanding of regional differences within the muscle. The current study highlights the many muscle fiber level changes that occur regionally within the muscle earlier than when gross atrophy is detectable in the supraspinatus at 2 weeks. Through histological fiber cross-sectional area analysis, the muscle pathology associated with rotator cuff tendon tear was evident within 1 week of injury, before detection of gross atrophy. Myofiber cross-sectional area is most affected 1 to 2 weeks after RCT, and the myofiber population returns to a normal distribution by 3 weeks in the MB, but remains smaller in the MTJ, suggesting a more persistent injury. These results are consistent with previous reports that muscle atrophy and cross-sectional area display the most deficit around 2 weeks and then return to control levels between 4 and 9 weeks in rodent models [18].

This is the first report, to our knowledge, showing differing patterns of degeneration along the length of the muscle. Corroborating this finding, recent studies have demonstrated that fibrosis and adipogenesis progress over time along the length of the muscle from lateral to medial [11]. In female animals, we also observed significant shifts in myofiber cross-sectional area 1 week after injury in the MTJ and near the intramuscular tendon (Fig. S8, Supplemental Information), suggesting that this spatial phenomenon is common to both male and female animals, although the relative differences between injured and uninjured cross-sectional area were less for female animals. However, for the remainder of the study, only male animals were used because we believed the relatively larger injury in the muscles (as shown by differences in myofiber size) of the male animals would facilitate investigation of underlying cellular mechanisms of de- and re-generation.

Myofiber degeneration as defined by decreased fiber area was localized to the lateral aspect of the muscle (MTJ) versus MB in lengthwise analysis and near the intramuscular tendon in cross-section (< 500um), while contralateral controls are not different regardless of location. The proximity of early degeneration and regeneration laterally near the tendon in the MTJ and in cross-section near the intramuscular tendon suggests that signals from the tendon (mechanical, biochemical, cellular) may drive degeneration, regeneration, or both [19]. Muscle atrophy is thought to be a result of mechanical unloading in RCT and nerve dysfunction [4], however, it is unclear whether this is related to the position of muscle fibers relative to the tendon. Since fibers in the MB have a differing pennation angle relative to fibers in the lateral region near the MTJ [16, 20], mechanical unloading may differentially affect these regions. Clinically, after RCT, the pennation angle of the muscle to the intramuscular tendon is altered dependent on the extent of tendon damage [21, 22].

Spatial analysis showed that regenerating eMHC-stained fibers were concentrated in the lateral MTJ area of the muscle and were more frequent near the intramuscular tendon. In embryonic muscle development, TGF-β super-family members such as *Bmp4* signaling at the tendon–muscle interface activates satellite cell proliferation preferentially near the tendon attachment point [19, 23]. In addition, in development and injury, satellite cells are found in higher frequency near the ends of the myofibers and support growth from the ends near the tendon–muscle interface [24]. These lines of evidence suggest that muscle regeneration may be more active at the ends of muscle fibers and closer to tendon–muscle junctions [23, 24], which may explain the increased eMHC + fibers and central nuclei in the MTJ and adjacent to the intramuscular tendon where bi-pennate muscle fibers attach. Previous work has shown the activation of satellite cells in the murine model of RCT [25], however, future analysis will be needed to assess spatial satellite cell activation in this injury model.

Fibrotic and fatty infiltration are thought to be hallmarks of RCT muscle-related clinical pathology [7]. In the current study, collagen was present between myofibers throughout the injured muscle, but not in the contralateral controls. In previous analysis of fibrous infiltration in this model using Masson’s Trichrome stain instead of antibody-based staining, significant fibrosis was observed from week 3–6 [15]. Here, by examining distinct regions of the muscle, collagen staining area was found to be elevated 1 week after RCT and remained elevated but did not further increase after the first week, consistent with recent reports in mouse [11]. In contrast to the spatiotemporal patterning of the degeneration and regeneration, collagen infiltration was similar between MTJ and MB near the intramuscular tendon, but was elevated in MTJ compared with MB in the region far from the intramuscular tendon. These data may indicate that differing mechanisms drive degeneration/regeneration versus collagen infiltration. Further studies will be needed to assess soluble and cellular signals that may be differentially represented near and far from the intramuscular tendon in this model.

Perilipin stained many cells in both injured and uninjured contralateral control that were frequently located near vascular structures. Perilipin + cells in this study did not show large lipid droplets or the characteristic globular shape of mature adipose tissue [26] and may be indicative of early fibro-adipogenic progenitor differentiation toward adipose lineage. Fatty infiltration is progressive and in clinical studies is shown to continue to increase with time in repaired or unrepaired RCT [27]. Here, Perilipin + cells were most elevated at the final timepoint assessed at 3 weeks post-RCT both near and far from the intramuscular tendon.

Inflammatory cells play a multifaceted role in muscle injury response [28–31] and therefore understanding the spatiotemporal localization of different inflammatory functional cells is an important step in examining their role in supraspinatus muscle pathology. Here we found that, following RCT, there are differing innate inflammatory cells recruited to the injured MTJ and MB, but not controls. In the MTJ, M1- and M2-like cells are elevated at all weeks, however by week 3, there are more M1- and M2-like near the intramuscular tendon, suggesting a balanced inflammatory environment followed by a later increase in pro-inflammatory cells at 3 weeks. Alternatively, in the MB, M1-like cells are dominant over M2-like cells throughout the timecourse, suggesting sustained inflammatory environment through 3 weeks. A limitation of this study is that M1- and M2- like cells were discriminated by only CD86 and CD206 surface markers rather than functional cytokine markers that might better define their role in the tissue. Further studies will be needed to address this point.

M1-like pro-inflammatory macrophages signal satellite cell proliferation, while M2-like macrophages signal myotube fusion [28]. In the MTJ, where M1- and M2-like cell populations were not different in the first 2 weeks, there were significantly more eMHC + fibers and centrally located nuclei indicating myoblast fusion. However, in the MB and far from the intramuscular tendon where cellular inflammation was primarily inflammatory M1-like cells, myofiber regeneration was significantly lower than in the MTJ. Highlighting the importance of macrophages in myofiber healing, we previously found that depletion of macrophages prior to RCT led to an increase in myofiber degeneration or an increase in small cross-sectional area fibers one week after injury [32]. Together, these data support an association between myofiber regeneration and the presence of differing subsets of macrophages.

Adaptive immune cells can also contribute to muscle pathology and repair, for example, regulatory T cells can potentiate muscle repair and frequently infiltrate muscle during the M2 macrophage-dominant repair phase [33]. In human RCT muscle samples and previous analysis in rodent models, total T-cells were not elevated relative to control muscles, suggesting T cells may not play a large role in the inflammatory response to RCT [32, 34]. By isolating distinct regions of interest within the muscle, here we were able to discern small, but significant, differences in T-cell infiltration compared with contralateral control. In RCT, T cells were elevated near the intramuscular tendon in both the MTJ and MB after 1 week, however at a much lower frequency than macrophages. The relatively low population of T cells following RCT may represent an opportunity for therapies targeting recruitment of pro-regenerative adaptive immune cells. 

Overall, this study demonstrates that the supraspinatus muscle undergoes regional changes after supraspinatus tendon injury, particularly that changes in muscle fiber size, markers of regeneration, and macrophage infiltration are more substantial in the lateral muscle near the myotendinous junction and radially near the intramuscular tendon. For pre-clinical models, these data suggest that experimental design must take into account the location in the muscle where data are collected. Moreover, the overall differences observed between the MTJ and MB suggest that further exploration of the role of tendon–muscle cross-talk in the context of RCT is warranted. Moving forward, these data suggest that novel therapeutic strategies that more specifically target a specific region may be required to achieve regeneration of the muscle after RCT. 

## Supplementary Information

Below is the link to the electronic supplementary material.

Supplementary file1 (DOCX 18520 KB)

## Data Availability

All relevant data generated or analyzed in this study can be obtained from the corresponding author upon reasonable request.
