# Supplementary material for: Supraspinatus Muscle Degeneration, Inflammation, and Regeneration Vary By Location in a Rat Model of Severe Rotator Cuff Tear
Source: Regen Eng Transl Med. 2026 Mar 19;12(2):787–99. doi: 10.1007/s40883-024-00343-3 (PMC13263273; doi:10.1007/s40883-024-00343-3)
Supplement: Supplementary file 1 — Supplementary file1 (DOCX 18520 KB) [file 40883_2024_343_MOESM1_ESM.docx]

**Supplemental Results and Discussion**

**Sex differences in myofiber cross-sectional area.**

To evaluate any possible sex differences in supraspinatus degeneration after RCT, muscle cross-sectional area was assessed in age-matched male and female one week after injury. Age-matching was chosen to ensure that the immunological age of the animal was the same since RCT is known to have a pathological inflammatory component.

Contralateral myofibers near the intramuscular tendon in female animals had a smaller mean area than in male animals (MTJ: (F) 903.8 $\pm$ 209.3 vs. (M) 1266 $\pm$ 279.4*; MB: (F) 1033.5 $\pm$625.2 vs. (M) 1301.9 $\pm$846.1*), which is not surprising given that the uninjured muscle weights were significantly different between the groups ((F) 324.6g vs. (M) 467.4g). Myofibers from female animals in the injured MTJ and MB near the intramuscular tendon were significantly smaller than contralateral control; however, the relative difference was smaller than in male animals (Fig. S8a-c). Comparison of fibers near and far from the intramuscular tendon showed that fibers near the tendon in injured muscle are significantly smaller than those far from the intramuscular tendon in both male and female animals (Fig. 2f-g, Fig. S8e-f). Muscle size is a confounding factor that make interpretation of differences between the sexes difficult; however, both male and female animals showed spatial differences (MTJ vs MB, near vs far from the intramuscular tendon) in muscle fiber cross-sectional area.

**Fig. S1 Muscle atrophy following rotator cuff tear**

Gross muscle weight after harvest of contralateral (white bars) or injured muscle (black bars) 1-3 weeks after tendon injury. n=5-6 per group. Data presented as mean ± S.D. with dots representing individual subjects. Data were analyzed by one way ANOVA and Tukey’s post-hoc test. * p<0.05.

**a**

**c**


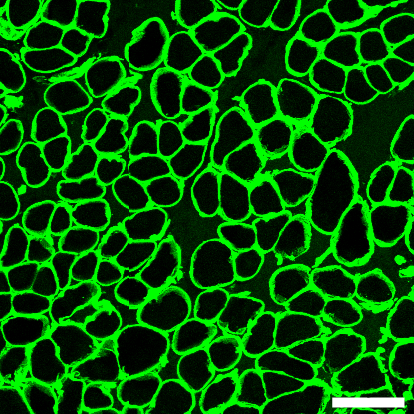

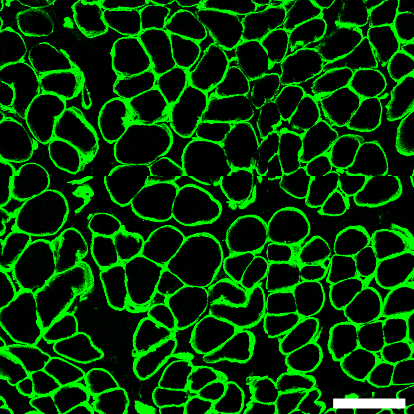

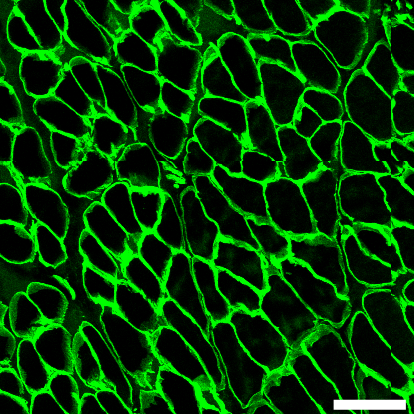


**Week 1**

**Week 2**

**Week 3**

**Laminin**

**Muscle Belly**

**(MB)**


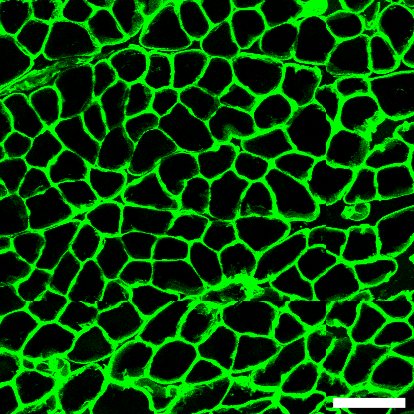

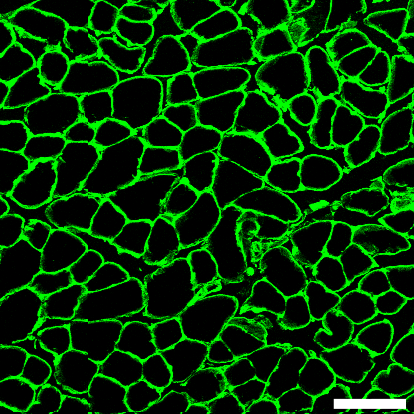

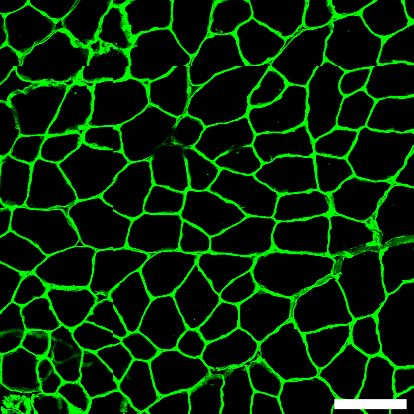


**Myotendinous Junction (MTJ)**

**Week 1**

**Week 2**

**Week 3**

**b**

**d**


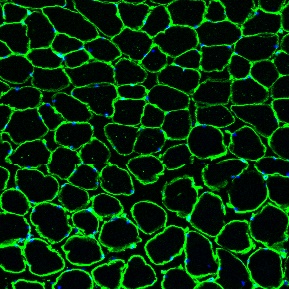


Near Intramuscular Tendon

Far from Intramuscular Tendon

**Contralateral Controls**


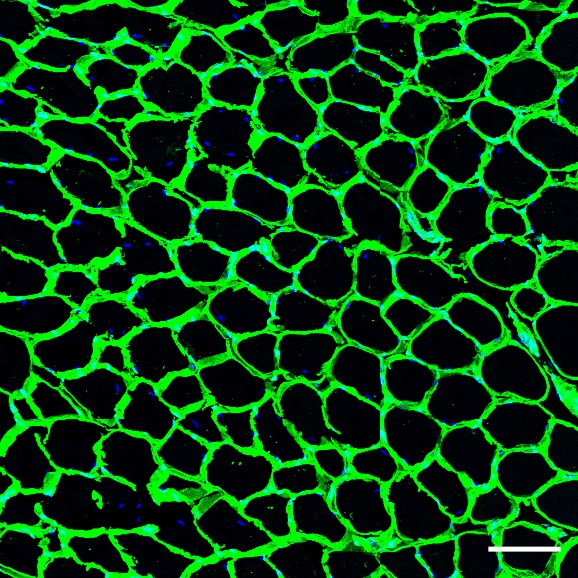

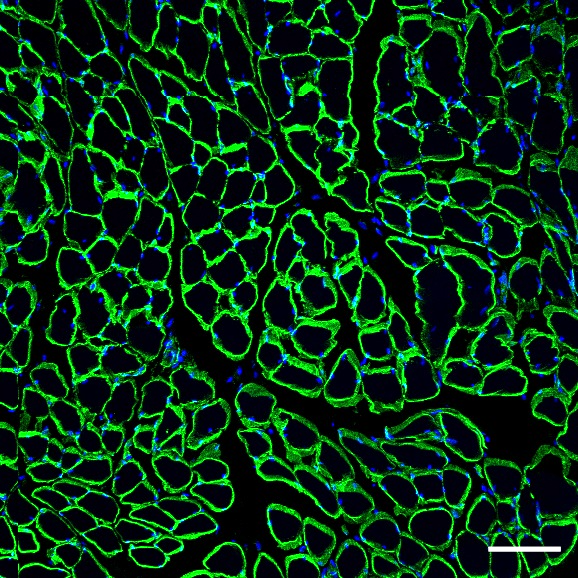

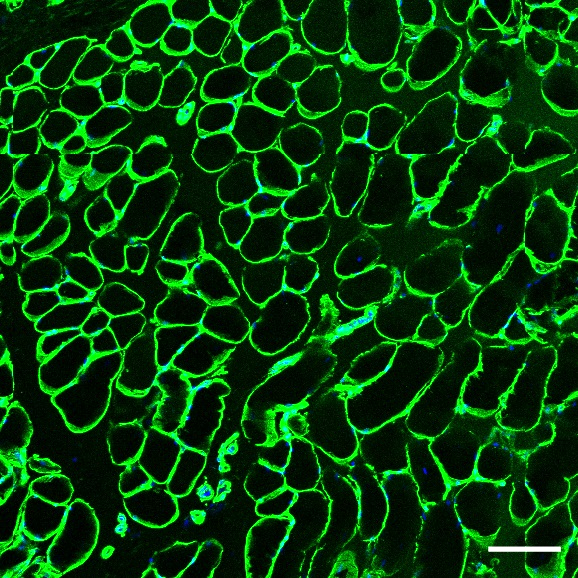

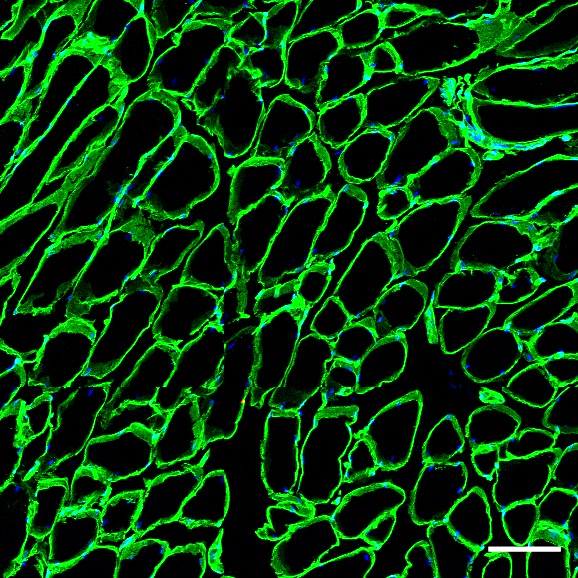

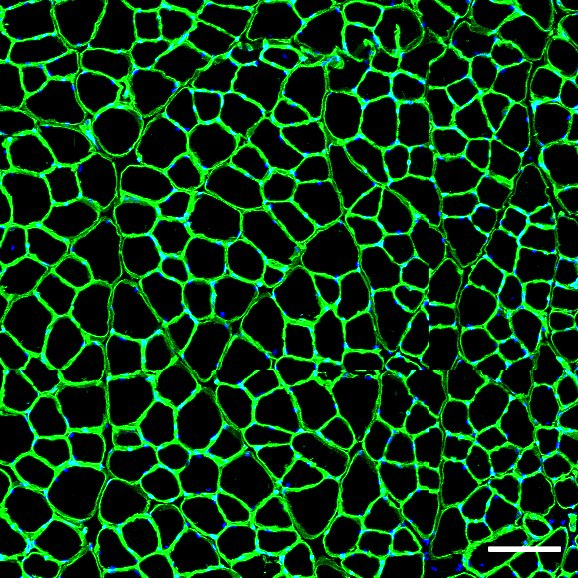


**Fig. S2 Images showing contralateral control muscles did not experience change in cross-sectional area after injury, regardless of location**

(**a-b**) Representative images of muscle fiber borders (laminin, green) in contralateral control (uninjured) muscle near the intramuscular tendon in the (**a**) myotendinous junction (MTJ) and the (**b**) muscle belly (MB). (**c-d**) Representative images far from the intramuscular tendon in the (**c**) MTJ or (**b**) MB. (scale bar 100μm)

**
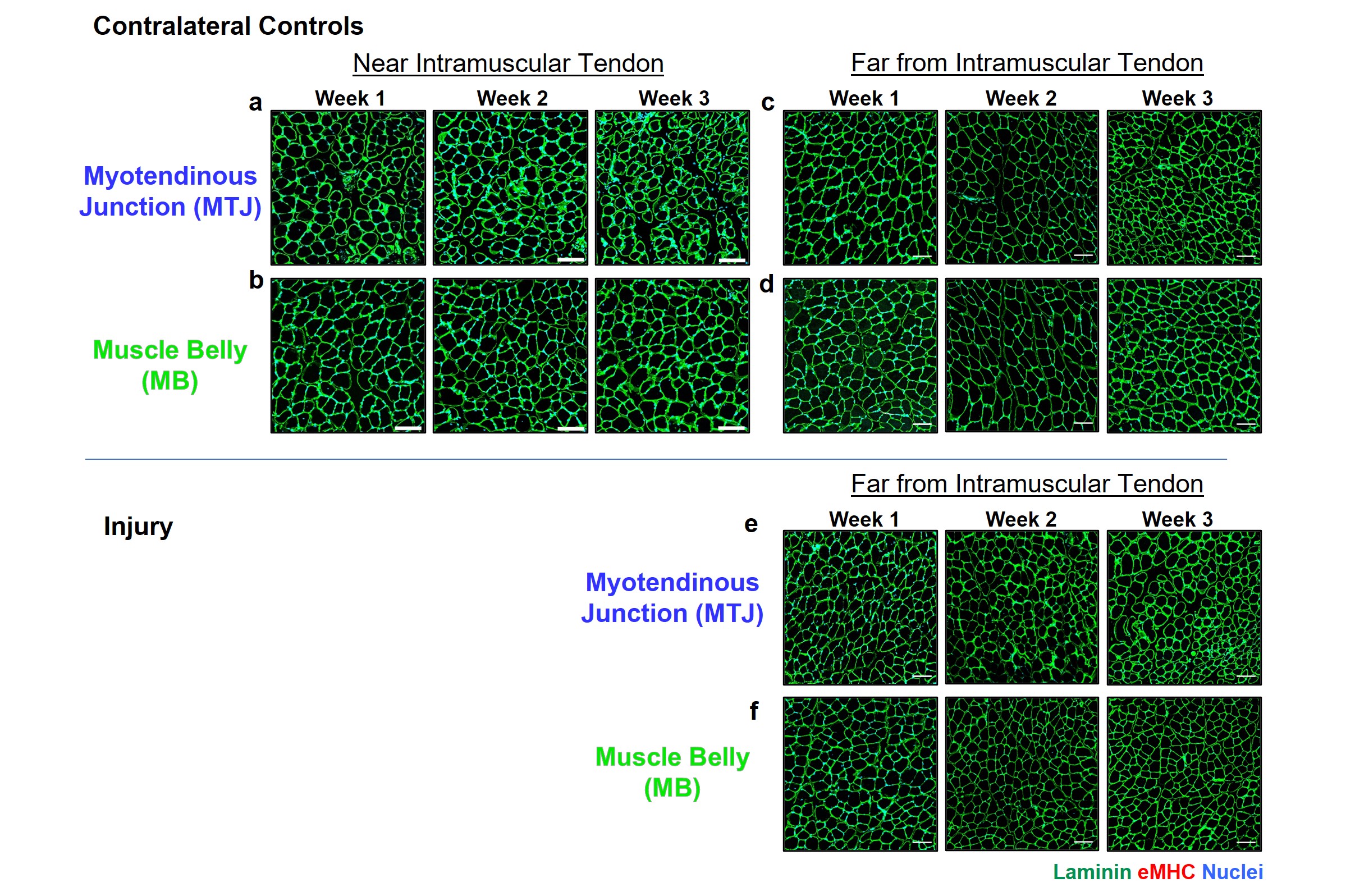
**

**Fig. S3 Regenerating myofibers are in low abundance in contralateral muscles and far from the intramuscular tendon**

(**a-b**) Representative images of regenerative marker staining in contralateral control (uninjured) muscles. Muscle fiber borders (laminin, green), embryonic myosin heavy chain (eMHC, red), and nuclei (Hoechst, blue) near the intramuscular tendon in the (**a**) myotendinous junction (MTJ) and the (**b**) muscle belly (MB). (**c-d**) Representative images far from the intramuscular tendon in the (**c**) MTJ or (**d**) MB. (**e-f**) Representative images in injured muscle far from the intramuscular tendon in the (**e**) MTJ or (f) MB (scale bar 100 μm)

**
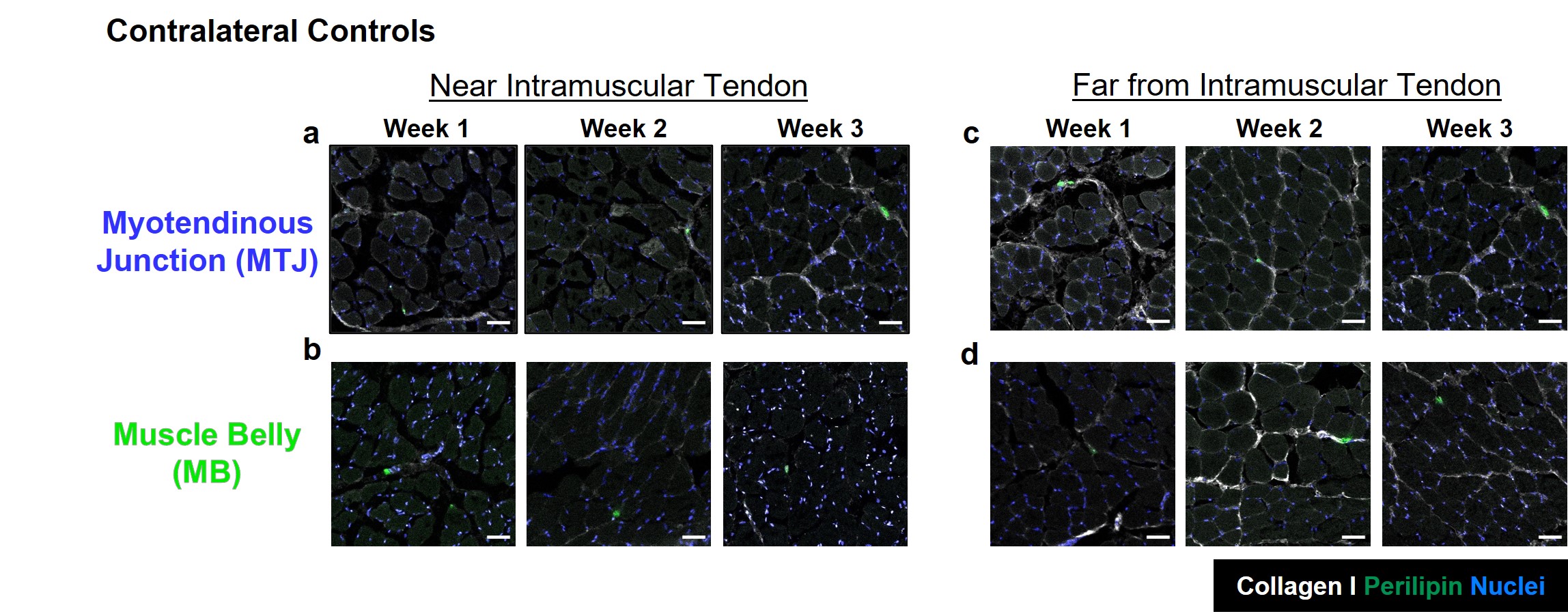
**

**Fig. S4 Contralateral control muscles do not have abundant collagen or perilipin staining**

(**a-b**) Representative images of fibrous (collagen I, white) and fatty (perilipin, green) infiltration and nuclei (Hoechst, blue) in contralateral control (uninjured) muscles near the intramuscular tendon in the (**a**) myotendinous junction (MTJ) and the (**b**) muscle belly (MB). (**c-d**) Representative images far from the intramuscular tendon in the (**c**) MTJ or (**d**) MB. (scale bar 50μm)


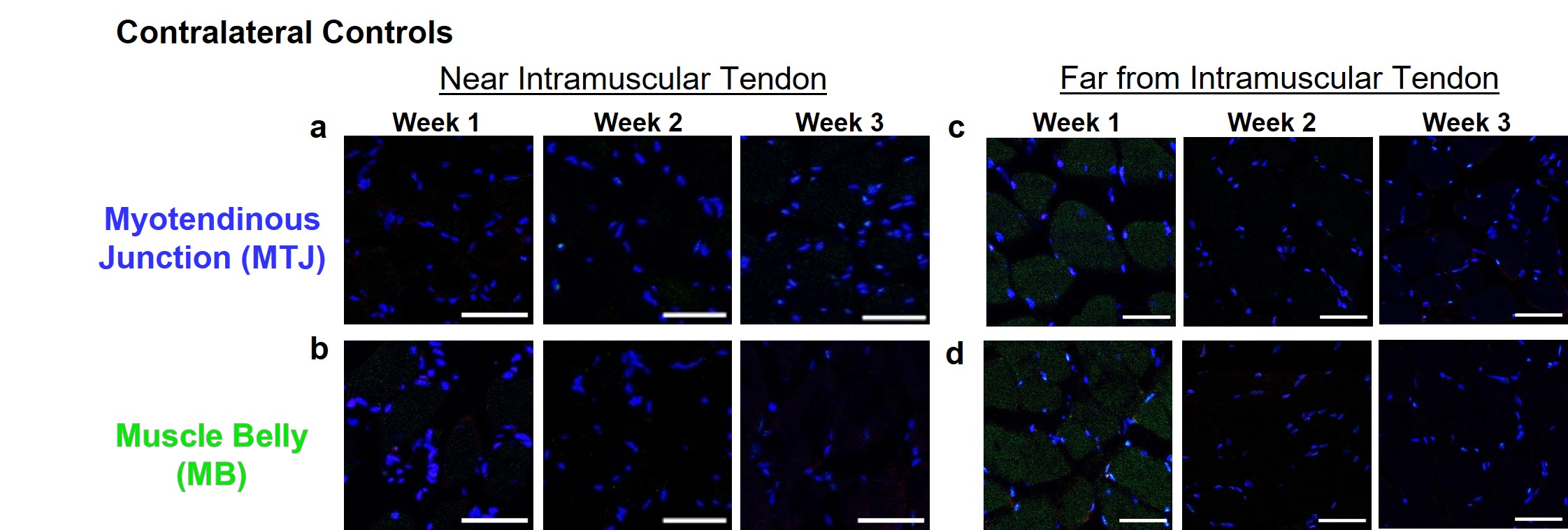


**Fig. S5 Contralateral control muscles have few macrophages**

(**a,b**) Representative images of macrophage markers in contralateral control (uninjured) muscles. M2-marker (CD206, green), M1-marker (CD86, red), and nuclei (Hoechst, blue) near the intramuscular tendon in the (**a**) myotendinous junction (MTJ) and the (**b**) muscle belly (MB). (**c,d**) Representative images far from the intramuscular tendon in the (**c**) MTJ or (**d**) MB. (scale bar 50μm)

**
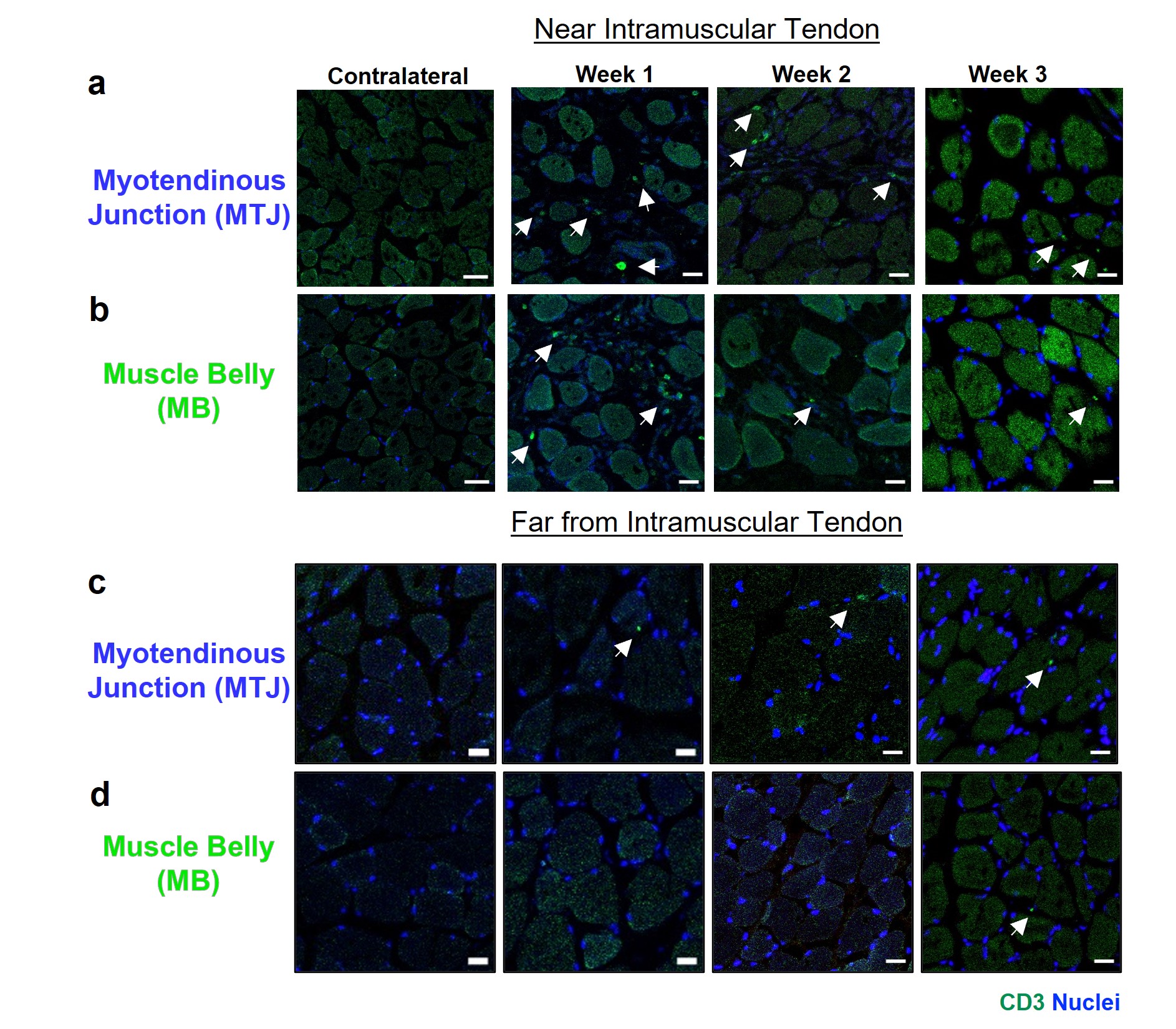
**

**Fig. S6 T cells are in low abundance in contralateral muscles and far from the intramuscular tendon**

(**a-b**) Representative images of CD3 staining in contralateral control (uninjured) muscles. T cells (CD3, green), and nuclei (Hoechst, blue) near the intramuscular tendon in the (**a**) myotendinous junction (MTJ) and the (**b**) muscle belly (MB). (**c-d**) Representative images far from the intramuscular tendon in the (**c**) MTJ or (**d**) MB. (scale bar 20μm)

**Fig. S7 T cell infiltration into the supraspinatus peaks 1 week after rotator cuff injury**

**a**

**b**

**Week 1**

**Week 2**

**Week 3**

**Contralateral**

**CD3 Nuclei**


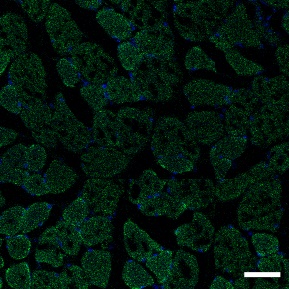

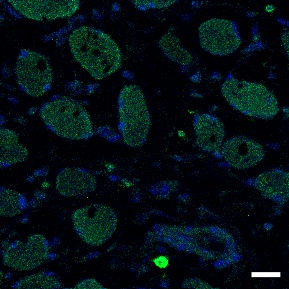

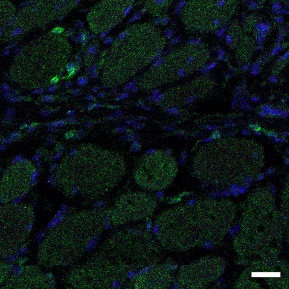

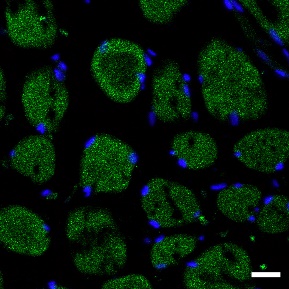

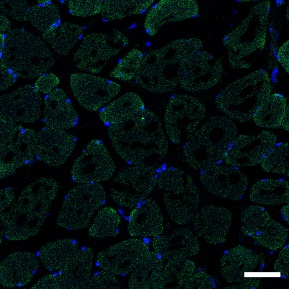

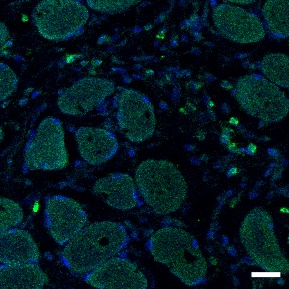

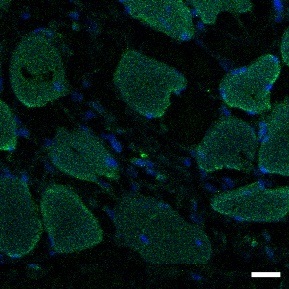

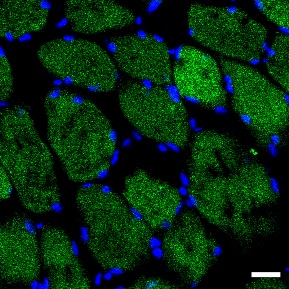


Near Intramuscular Tendon

**Muscle Belly**

**(MB)**

**Myotendinous Junction (MTJ)**


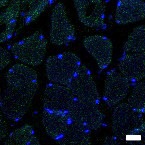

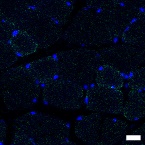

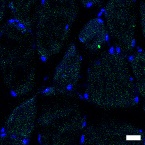

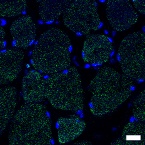


**Muscle Belly**

**(MB)**

**Myotendinous Junction (MTJ)**

**c**

**d**

Far from Intramuscular Tendon


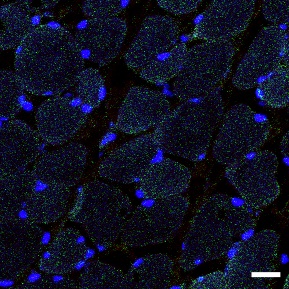

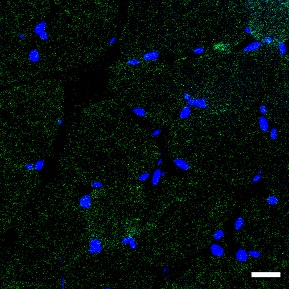

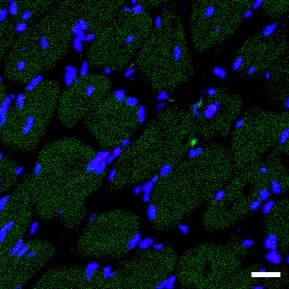

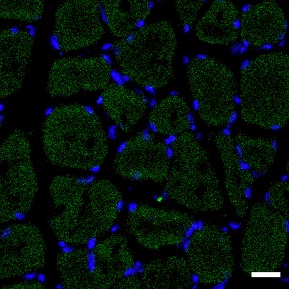


Representative images of T cells in the supraspinatus muscle (**a-b**) near the intramuscular tendon in the (**a**) MTJ or (**b**) MB; and (**c-d**) far from the intramuscular tendon in the (**c**) MTJ or (**d**) MB (CD3: green; nuclei: blue; white arrow: T cells; scale bar 20 μm)

**Fig. S8 Female rats have less change in myofiber cross-sectional area than male rats 1 week after rotator cuff injury**(**a,b**) Distribution of cross-sectional myofiber area in the MTJ near the intramuscular tendon in (**a**) male or (**b**) female age-matched rats 1 week after rotator cuff injury. (**c,d**) Cross-sectional myofiber area distribution in the MB near the intramuscular tendon in (**c**) male or (**d**) female age-matched rats. (**e, f**) Myofiber area in the MTJ >500um from the intramuscular tendon in (**e**) male or (**f**) female age-matched rats. (**g, h**) Myofiber area in the MB >500um from the intramuscular tendon in (**g**) male or (**h**) female age-matched rats. (2-way ANOVA, with post hoc multiple comparisons test by bin; # p<0.05 vs. contralateral; n=5-7)
